# Estigmatización y pobreza: pueblos indígenas del sureste de México frente al síndrome de inmunodeficiencia adquirida

**DOI:** 10.1590/S0104-59702024000100059

**Published:** 2024-11-04

**Authors:** América Molina del Villar

**Affiliations:** i Centro de Investigaciones y Estudios Superiores en Antropología Social. Ciudad de México – México. avillar@ciesas.edu.mx

El virus de la inmunodeficiencia humana (VIH) que provoca el síndrome de inmunodeficiencia adquirida (sida) emergió cuando gran parte de los países industrializados habían relajado las medidas de control de las enfermedades infecciosas. En 1980, gran cantidad de antibióticos, sulfas, penicilinas y vacunas habían vencido a estos padecimientos. Marcos Cueto (2002) refiere que este optimismo se derrumbó al emerger la pandemia del sida. Médicos, pacientes y activistas se enfrentaron a la corriente del neoliberalismo para hacer del sida un tema prioritario de la salud pública mundial. La enfermedad surgió en un contexto mundial de crisis económica y, en relación con México, en medio de la reestructuración de sus políticas económicas. El país enfrentaba una coyuntura crítica de devaluaciones, recortes sistemáticos al gasto público y en particular al gasto social. En México, desde la década de 1980 se declaró la suspensión de pagos a los acreedores externos, factores que incidieron negativamente en la respuesta ante la pandemia debido a las políticas de austeridad en el gasto en salud. Entre 1983 y 1988 los programas de atención preventiva en las principales instituciones de salud pública no alcanzaron el 3% del gasto de salud y seguridad social. Los primeros casos de sida en México se notificaron en 1983. A partir de este año se fue incrementando el número de contagios.

A 40 años de aparición del sida en el planeta, disponemos del libro *Pueblos indígenas ante la epidemia del VIH: políticas, culturas y prácticas de la salud en Chiapas y Oaxaca*, de Rubén Muñoz Martínez (2023), que nos ofrece un estudio robusto, producto de una investigación de cerca de 10 años. El libro consta de introducción, siete capítulos, conclusiones y bibliografía. En 422 páginas, el autor examina el impacto de la epidemia en el sur del país, Chiapas (Ocosingo) y Oaxaca, poblaciones con altos niveles de marginación y pobreza. El trabajo de campo se realizó entre 2017 y 2018, periodo importante debido a la crisis en los sistemas de salud pública producto de las políticas neoliberales. Entre 2010 y 2015 la mayor mortalidad por VIH convergió hacia grandes concentraciones demográficas, ciudades fronterizas y turísticas. Desde el enfoque de la antropología médica crítica, Rubén Muñoz analiza las múltiples afectaciones del sida en estas poblaciones víctimas a su vez de la marginación, pobreza, migración y violencia por narcotráfico. En la obra se da voz a las víctimas de la enfermedad, así como al personal de salud que enfrenta la falta de recursos. Las poblaciones examinadas se encuentran alejadas de laboratorios y hospitales de altas especialidades. El diagnóstico y atención médica tardía ha provocado un crecimiento exponencial de enfermos y muertos.

El sida es todavía una enfermedad temida y encerrada en infinitos misterios, en cuanto a su origen y clínica. El hecho de que, para fines prácticos, se trate de un padecimiento de transmisión sexual ha determinado que esté rodeado de mitos sobre su contagiosidad. Lo anterior explica el afán de negar el fenómeno, después de buscar culpables y estigmatizar poblaciones (Sontag, 1989). Estas reacciones han sido visibles durante la peste, la viruela, el tifo, la sífilis, la influenza y la covid-19. La reconstrucción detallada de los testimonios expuestos en el libro de Rubén Muñoz visibiliza estas manifestaciones de estigmatización y miedo. El acercamiento metodológico de la obra se apoya en entrevistas a profundidad y en observaciones. El autor realizó más de cincuenta entrevistas en personas de entre 20 y 50 años. El enfoque comparativo de poblaciones indígenas en Chiapas y Oaxaca es muy valioso, en virtud de que identifica similitudes y diferencias con respecto a la atención médica, condiciones de pobreza, migración, discriminación y rechazo social. También el estudio atraviesa el tema de género, destacando la mayor vulnerabilidad de las mujeres a la infección; por ejemplo, el menor uso de condón en sus relaciones sexuales. Del mismo modo, la detección tardía de la enfermedad por temor al rechazo y discriminación. Cabe destacar escenarios de contraste en ambos estudios de caso; por ejemplo, la organización social de apoyo a personas afectadas por la pandemia en Oaxaca, en contraste con Ocosingo. Si alguna enseñanza ha dejado esta pandemia en el mundo es la visibilidad de la comunidad *gay* y sus plenos derechos respecto a la tolerancia y a la salud. También la apertura en el ámbito privado y público de la educación sexual exenta de tabúes, impartida desde la infancia, constituyendo un tema de salud pública, es otra de las enseñanzas que ha aportado la pandemia. Sin embargo, en el caso de las comunidades analizadas por Rubén Muñoz, esta situación está lejos de cristalizarse.

El estudio sobre el impacto del sida en Chiapas y Oaxaca atraviesa aspectos relacionados con la etnicidad, racismos, migración, pobreza, vulnerabilidad femenina, sexualidad y concepciones culturales. También aporta estadísticas sobre morbilidad y mortalidad en pueblos indígenas, donde prevalece un fuerte subregistro, carencia que impide una actuación gubernamental oportuna de prevención y atención médica. De ahí la importancia de adentrarse en las historias de vida de las víctimas de la pandemia, que no solo sufren las secuelas clínicas de la enfermedad, el dolor y la muerte, sino también la discriminación de la familia, de la sociedad, el abandono y desinterés de las autoridades de salud. Así, como señala el autor, la condición étnica articulada, con variables como el género y clase social, se constituyen en efectos de vulnerabilidad social ante la pandemia. Rubén Muñoz señala la relevancia de disponer más estudios a nivel cuantitativo y cualitativo sobre prevalencia, impacto y etnicidad del sida, temas ausentes en los estudios de epidemiología del país. La antropología, la historia y las ciencias sociales constituyen disciplinas valiosas para la epidemiología y salud pública del pasado y presente. El libro constituye un aporte significativo.
